# Oxidized Dextran/Carboxymethyl Chitosan Dynamic Schiff-Base Hydrogel for Sustained Hydrogen Sulfide Delivery and Burn Wound Microenvironment Remodeling

**DOI:** 10.3390/pharmaceutics18030370

**Published:** 2026-03-17

**Authors:** Zhishan Liu, Ying Zhu, Zhuoya Ma, Xuyang Ning, Ziqiang Zhou, Jinchang Liu, Youfu Xie, Gang Li, Ping Hu

**Affiliations:** 1 Department of Burns & Plastic Surgery, Guangzhou Red Cross Hospital, Faculty of Medical Science, Jinan University, Guangzhou 510006, China; 1210381703@qq.com (Z.L.); 17320688325@163.com (Z.M.); 17670960705@163.com (X.N.); zzq970114@163.com (Z.Z.); njljc1205@163.com (J.L.); xiett@vip.sina.com (Y.X.); 182340409@qq.com (G.L.); 2 State Key Laboratory of Bioactive Molecules and Druggability Assessment, Jinan University, Guangzhou 510006, China; 2139544624@qq.com; 3 College of Pharmacy, Jinan University, Guangzhou 510006, China

**Keywords:** oxidized dextran, carboxymethyl chitosan, hydrogen sulfide delivery, self-healing hydrogel, burn wound healing, microenvironment remodeling

## Abstract

**Background**: Polysaccharide-based dynamic hydrogels are promising for wound management due to their biocompatibility, injectability, and tunable biofunctionality. The integration of therapeutic gasotransmitter donors offers a strategy to modulate the wound microenvironment. **Objectives**: This study aimed to develop an injectable, self-healing carbohydrate hydrogel capable of sustained hydrogen sulfide (H_2_S) release for burn wound therapy, and to evaluate its physicochemical properties, in vivo efficacy, and mechanism of action. **Methods**: A dynamic hydrogel (ACMOD) was fabricated via Schiff-base crosslinking between oxidized dextran (OD) and carboxymethyl chitosan (CMCS), incorporating the H_2_S donor 5-(4-hydroxyphenyl)-3H-1,2-dithiole-3-thione (ADT-OH). Rheological and recovery tests characterized its mechanical and self-healing properties. Efficacy and mechanisms were assessed in a rat full-thickness burn model, analyzing wound closure, histology, oxidative stress, macrophage polarization, angiogenesis, and collagen deposition. **Results**: ACMOD exhibited shear-thinning, rapid self-healing, and strong tissue adherence. Sustained H_2_S release from ACMOD significantly accelerated wound closure and improved tissue regeneration compared to controls. Mechanistically, H_2_S attenuated oxidative stress, promoted a pro-regenerative M2 macrophage phenotype, enhanced angiogenesis via VEGF upregulation, and fostered organized collagen deposition and extracellular matrix remodeling. **Conclusions**: This work demonstrates a versatile, carbohydrate-based dynamic hydrogel platform that synergizes polymer network dynamics with bioactive H_2_S delivery to effectively promote burn wound healing. The findings underscore the potential of polysaccharide hydrogels with integrated gasotransmitter release for regenerative therapy and biomaterials applications.

## 1. Introduction

Skin, the body’s largest organ, serves as the primary barrier against external insults [1]. Burn injuries, particularly deep partial-thickness (deep second-degree) and full-thickness burns, cause severe disruption of this barrier, triggering a complex cascade of pathophysiological events [2]. Unlike typical wounds, burn wound healing is often protracted and problematic, characterized by persistent and excessive inflammation, elevated oxidative stress, increased susceptibility to bacterial infection, impaired neovascularization, and dysfunctional fibroblast activity leading to aberrant collagen deposition [3]. These intertwined pathological factors frequently result in delayed healing, hypertrophic scarring, and long-term functional impairments, posing a significant clinical challenge [4].

Conventional burn dressings, such as petroleum jelly gauze or silicone sheets, primarily function as passive coverings. Although they provide a basic physical barrier, they lack the capacity to actively intervene and modulate the wound microenvironment, falling short of the requirements for high-quality regenerative healing in modern precision medicine [5]. Consequently, the development of “smart” dressings endowed with intrinsic bioactivity and capable of dynamically responding to and actively promoting wound repair has become a central focus in biomaterials and regenerative medicine research [6]. Among various candidates, hydrogel dressings are particularly attractive due to their three-dimensional hydrophilic networks, which ensure moisture retention, excellent biocompatibility, and versatility as delivery vehicles for therapeutic agents [7].

Recent advances in gasotransmitter biology have revealed the critical roles of endogenous gaseous signaling molecules, including nitric oxide (NO), carbon monoxide (CO) [8], and hydrogen sulfide (H_2_S) [9,10], in tissue repair and regeneration. H_2_S, once regarded solely as a toxic environmental gas, is now recognized as a pivotal regulator of diverse cellular functions [11,12,13]. In skin wound healing, H_2_S exerts multiple biological effects: it suppresses excessive inflammation by inhibiting key signaling pathways such as NF-κB [14], alleviates oxidative stress by activating endogenous antioxidant defenses, including the Nrf2 pathway [15], promotes angiogenesis by stimulating endothelial cell proliferation, migration, and tube formation [16], and modulates fibroblast behavior, supporting collagen synthesis while potentially preventing pathological scarring [17].

To harness these therapeutic benefits, exogenous H_2_S donors have been developed, among which ADT-OH (5-(4-hydroxyphenyl)-3H-1,2-dithiole-3-thione) stands out as a well-established and reliable tool. This donor is particularly valued for its favorable release kinetics, high chemical stability, and superior H_2_S yield compared to traditional sulfide salts [18]. Despite these advantages, the clinical translation of gas therapy for wound healing remains significantly constrained by the inherent difficulty of achieving localized, sustained, and controllable delivery of gaseous mediators to the wound site [19]. Systemic administration often leads to off-target effects and fails to maintain therapeutically relevant concentrations over time. Therefore, engineering an effective delivery system capable of efficiently loading, protecting, and releasing ADT-OH in a controlled manner directly into the wound microenvironment is essential for fully realizing its therapeutic potential and advancing gas therapy toward clinical application.

Although injectable hydrogels can function as drug carriers, their crosslinked networks are often static and irreversible [20]. In these conventional systems, structural integrity and functionality are entirely dependent on maintaining this permanent network. When subjected to mechanical stress in the dynamic in vivo environment, these hydrogels can undergo irreversible fracture, leading to loss of structural integrity, premature drug leakage, and dressing failure, which may necessitate repeated replacement [21]. This limitation is particularly pronounced for burn wounds located near joints or other highly mobile anatomic sites. Thus, an ideal wound dressing should not only exhibit good initial conformability but also possess the ability to autonomously repair structural damage, thereby maintaining long-term barrier function and controlled drug release [22].

To address these inherent drawbacks of static networks, self-healing hydrogels have been developed [23]. Unlike their irreversible counterparts, the functionality of these advanced materials does not depend on preserving a permanent, static network. Instead, they achieve high efficiency precisely through the absence of one. Their design relies on dynamic and reversible crosslinking interactions, such as Schiff base bonds [24], boronic ester bonds [25], and hydrogen bonds [26]. This dynamic chemistry provides a fundamentally different mechanism for maintaining integrity: upon mechanical damage, these reversible bonds preferentially dissociate to dissipate energy, thereby preventing catastrophic network failure. Once the stress subsides, the bonds readily re-form at the damaged interface, enabling spontaneous restoration of macroscopic structure and mechanical performance [27]. In essence, self-healing hydrogels remain efficient not by resisting damage through a permanent structure, but by embracing dynamicity—using reversible bonds that break to absorb energy and then reform to heal. Among these systems, Schiff-base hydrogels formed through reactions between aldehyde and amino groups are particularly attractive for wound healing applications due to their mild reaction conditions (often catalyst-free), excellent biocompatibility, and rapid gelation kinetics [28].

**Scheme 1:**
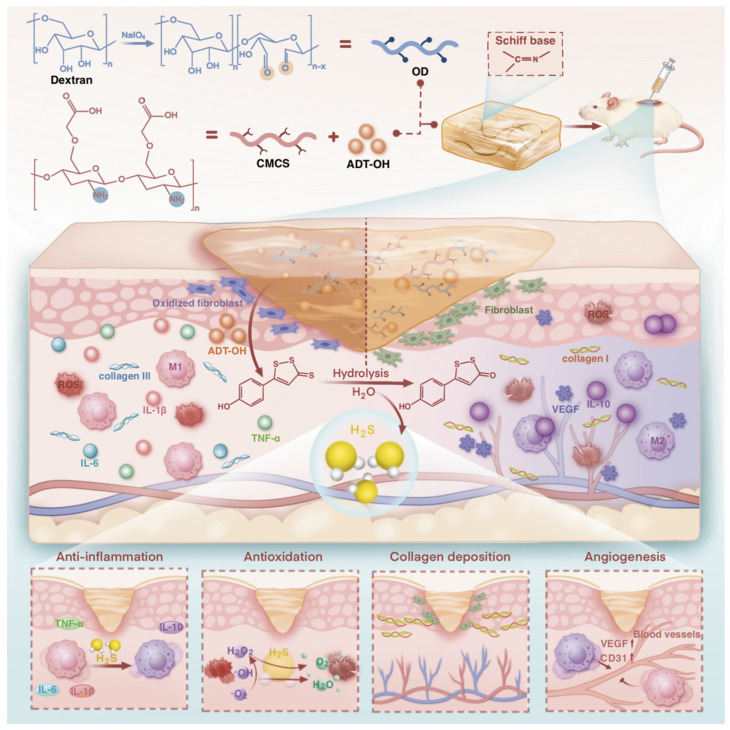
Schematic illustration of the preparation and application of the ACMOD hydrogel, which promotes burn wound healing by alleviating inflammation and oxidative stress while enhancing angiogenesis and collagen remodeling.

Here, we propose an integrated treatment strategy that combines self-healing hydrogel properties with H_2_S-mediated gas therapy for burn wound healing (Scheme 1). To achieve this, we designed a Schiff base-crosslinked hydrogel based on carboxymethyl chitosan (CMCS) and oxidized dextran (OD) as an intelligent delivery vehicle for the H_2_S donor ADT-OH. The dynamic and reversible nature of the imine bonds confers not only autonomous self-healing capability but also inherent pH responsiveness [29], enabling on-demand ADT-OH release in response to the mildly acidic microenvironment characteristic of burn wounds [30]. Sustained H_2_S release subsequently acts as a potent biological signal to regulate inflammation, oxidative stress, and angiogenesis [31]. In this study, we systematically developed and evaluated this hydrogel platform, investigating its self-healing behavior, pH-responsive H_2_S release profile, and therapeutic efficacy through comprehensive in vitro and in vivo experiments. This work not only provides an effective strategy for localized and sustained delivery of ADT-OH but also broadens the application of self-healing hydrogels in gasotransmitter-based therapies for wound repair.

## 2. Materials and Methods

### 2.1. Materials

CMCS (MW ~240 kDa) with a 90% substitution degree of Carboxymethyl groups and a deacetylation degree of 90%, Dextran (MW ~70 kDa), sodium periodate (NaIO_4_) and crystal violet were purchased from Shanghai Macklin Biochemical Co., Ltd. (Shanghai, China). ADT-OH was purchased from Shanghai Bide Medical Technology Co., Ltd. (Shanghai, China). Rabbit red blood cells were purchased from Guangzhou Hongquan Biotechnology Co., Ltd. (Guangzhou, China). D_2_O was purchased from Shanghai Jizhi Biochemical Technology Co., Ltd. (Shanghai, China). Paraformaldehyde (4%) was purchased from Wuhan Xavier Biotechnology Co., Ltd. (Wuhan, China). DMEM-F12 (1:1) medium, fetal bovine serum (FBS) and penicillin-streptomycin were purchased from Thermo Fisher Biochemicals Co., Ltd. (Waltham, MA, USA). MTT cell proliferation and cytotoxicity assay kits and calcein/PI cell viability/cytotoxicity assay kits were purchased from Shanghai Biyuntian Biotechnology Co., Ltd. (Shanghai, China). Matrigel was purchased from Corning Incorporated (Corning, NY, USA). All other reagents were commercially pure for analysis.

### 2.2. Synthesis of Oxidized Dextran

Oxidized Dextran (OD) was synthesized according to a reported method [32]. Dextran (4 g, equivalent to 0.02 mol of anhydroglucoside units) was dissolved in 320 mL of ultrapure water with stirring at 37 °C until complete dissolution, followed by cooling to room temperature. NaIO_4_ (4.2 g, 0.02 mol) was dissolved in 80 mL of ultrapure water under light-protected conditions, and this solution was slowly added to the dextran solution. The reaction mixture was magnetically stirred in the dark at room temperature for 24 h. Subsequently, the product was dialyzed against deionized water using dialysis tubing (MWCO 8000, Secoma Biotechnology Co., Ltd., Beijing, China) for 3 days, with frequent water changes, and then lyophilized to obtain the OD.

### 2.3. Characterization of OD

The chemical structures of dextran and OD were analyzed by Fourier-transform infrared (FT-IR) spectroscopy (JASCO, Tokyo, Japan). The oxidation degree of OD was determined using the hydroxylamine hydrochloride titration method. ^1^H nuclear magnetic resonance (^1^H NMR) spectra of dextran and OD were recorded on an Avance III 400 MHz spectrometer (Bruker Technology Co., Ltd., Beijing, China) using D_2_O as the solvent.

### 2.4. Preparation of Hydrogels

Blank Hydrogel (CMOD): 500 mg OD was dissolved in 10 mL of phosphate-buffered saline (PBS, 10 mM, pH 7.4) by vortexing to obtain a 5.0% (*w*/*v*) OD solution. Separately, carboxymethyl chitosan (CMCS) solutions at concentrations of 2.5%, 5.0%, and 7.5% (*w*/*v*) were prepared. The OD solution was then mixed with each CMCS solution at a fixed volume ratio of 1:6 (OD:CMCS) to form blank hydrogels, designated as CMOD hydrogels. ADT-OH-Loaded Hydrogel (ACMOD): ADT-OH was uniformly dispersed into the CMCS solutions. These dispersions were subsequently mixed with the OD solution at the same 1:6 volume ratio (OD:CMCS dispersion) to form drug-loaded hydrogels, designated as ACMOD hydrogels. The chemical structures of lyophilized CMOD and ACMOD hydrogels were analyzed using FT-IR spectroscopy. The gelation time was determined by the vial inversion method. Three independent experiments were conducted (n = 3).

### 2.5. Self-Healing Property of the Hydrogel

To demonstrate the macroscopic self-healing capability, two identical hydrogel discs were prepared using a circular mold. One disc was stained with Rhodamine B and the other with Methyl Orange. Each disc was carefully bisected with a clean blade. A half stained with one dye was then placed in contact with a half stained with the other dye, reassembling a complete disc. Without applying any external force, the reassembled hydrogel was left undisturbed for 2 h at ambient conditions, after which the interfacial integration was visually examined to assess the healing outcome.

### 2.6. Scanning Electron Microscopy (SEM) Analysis

For morphological characterization, the prepared hydrogels were frozen at −80 °C and subsequently lyophilized. The lyophilized samples were carefully sectioned into thin slices, and any debris was gently removed. The slices were then sputter-coated with gold and examined under a scanning electron microscope to observe their internal microstructure.

### 2.7. Rheological Characterization

The rheological properties of the hydrogel were evaluated using a rotational rheometer equipped with a parallel-plate geometry (gap: 0.3 mm) at 37 °C. To examine shear-thinning behavior, steady-state viscosity was measured over a shear rate range of 0.1–1000 s^−1^. Time-dependent stability was assessed by monitoring the storage modulus (G′) and loss modulus (G″) during a frequency sweep from 0.1 to 10 rad/s over a defined period. The linear viscoelastic region (LVR) was determined via an amplitude sweep at a fixed frequency of 1 Hz, with strain varying from 0.1 to 1000%. To probe microscopic self-healing behavior, alternating strain step tests were performed by applying 1% strain (60 s) followed by 600% strain (60 s) repeatedly, while recording G′ and G″ throughout the cycles.

### 2.8. Swelling Behavior of the Hydrogel

The swelling characteristics of the hydrogel were evaluated as follows. First, the hydrogel was lyophilized and weighed to obtain its initial dry weight (W_0_). It was then immersed in PBS at room temperature. At predetermined time points (0, 0.5, 1, 2, 4, 6, 8, 12 and 24 h), the hydrogel was carefully removed, gently blotted to remove surface liquid, and weighed to obtain the wet weight (W_t_). Three independent experiments were conducted (n = 3). The swelling ratio (SR) was calculated according to the following Equation (1):

Swelling ratio (%) = (W_t_ − W_0_)/W_t_ × 100% (1)

where W_t_: the moisture content of the hydrogel at t hours, W_0_: the initial dry weight of the hydrogel.

### 2.9. In Vitro Drug Release from ACMOD Hydrogel

The in vitro release of ADT-OH from the ACMOD hydrogel was studied as follows. A hydrogel sample containing 452.7 μg of ADT-OH (total volume 350 μL) was placed into 10 mL of release medium (PBS with 0.1% SDS) and incubated at 37 °C under mild agitation. At predetermined time points (0.5, 1, 2, 4, 8, 12, 24, 36, 48 and 72 h), 1 mL of the release medium was withdrawn and replaced with an equal volume of fresh medium. The amount of released ADT-OH was determined by measuring the absorbance at 436 nm. A total of three independent trials were carried out (n = 3). The cumulative release percentage was calculated according to the following Formula (2):

(2) $$\mathrm{Cumulative}\;\mathrm{release}\;(\%)\;=\;(\mathrm{Cn}\;\times \;V1\;+\sum_{i=1}^{n-1}\mathrm{Ci}\;\times \;V2)/m\;\times \;100\%$$

where Cn: concentration measured at the nth sampling point, V1: release medium volume, Ci: concentration measured at the *i*th sampling point, V2: Sampling volume, m: the initial weight of ADT-OH in the hydrogel.

### 2.10. H_2_S Release from ACMOD Hydrogel

The release of hydrogen sulfide (H_2_S) from the ACMOD hydrogel was monitored using the methylene blue assay, and three independent experiments were conducted (n = 3). Briefly, a reaction mixture (2 mL) was prepared containing a specified amount of the hydrogel (loaded with ADT-OH), 40 mM zinc acetate (Zn(OAc)_2_), 100 mM hydrogen peroxide, and carboxyanhydrase (CA, concentration specified in mg·mL^−1^). At predetermined time points (0, 3, 6, 9, 12, 24, 48, 72 and 96 h), 50 μL of ferric chloride solution (30 mM in 1.2 M HCl) and 50 μL of N,N-dimethyl-p-phenylenediamine dihydrochloride solution (20 mM in 7.2 M HCl) were added. After incubation in the dark for 4 h, the absorbance at 674 nm was recorded. The H_2_S concentration was determined using a calibration curve established with sodium sulfide standards.

### 2.11. Hemolysis Assay

The hemolytic potential of the hydrogels was evaluated using a rabbit red blood cell (RBC) suspension, and three independent experiments were conducted (n = 3). Briefly, 1 mL of a 5% (*v*/*v*) rabbit RBC suspension was added to each of the following samples: 1 mL of homogenized ACMOD hydrogel, 1 mL of homogenized CMOD hydrogel, 1 mL of PBS (10 mM, pH 7.4) as a negative control, and 1 mL of 2% Triton X-100 solution as a positive control. The mixtures were incubated at 37 °C in a thermostatic shaker for 1 h. Subsequently, they were centrifuged at 2000 rpm and 0–4 °C for 10 min. From each tube, 100 μL of supernatant was transferred to a 96-well plate, and the absorbance was measured at 540 nm. The hemolysis percentage was calculated according to the following Formula (3):

Hemolysis (%) = ((OD_sample_ − OD_PBS_)/(OD_Triton X−100_ − OD_PBS_)) × 100% (3)

where OD_sample_: the optical density of the sample group, OD_PBS_: the optical density of the negative control group, OD_Triton X−100_: the optical density of the positive control group.

### 2.12. Cell Culture

Human umbilical vein endothelial cells (HUVECs), obtained commercially from Shanghai Sai Baikang Biotechnology Co., Ltd. (Shanghai, China), were cultured as follows. The growth medium consisted of DMEM/F-12 supplemented with 10% FBS and the antibiotics penicillin (100 U/mL) and streptomycin (100 µg/mL). Cells were incubated at 37 °C in a 5% CO_2_ environment with saturated humidity.

### 2.13. Preparation of Hydrogel Extract

To prepare the hydrogel extract, 4 mL of blank hydrogel was immersed in 40 mL of DMEM/F-12 basal medium supplemented with 1% penicillin-streptomycin (antibiotic-antimycotic). After incubation at 37 °C with shaking at 100 rpm for 24 h, the mixture was centrifuged at 5000 rpm for 10 min. The supernatant was collected and sterilized by filtration through a 0.22 μm filter to obtain the hydrogel extract.

### 2.14. MTT Assay

Cell proliferation was evaluated using the MTT method. After 24 h of exposure to the hydrogel extract, the original medium was discarded. Then, in the dark, each well (including both control and treated samples) received 90 μL of DMEM/F-12 basal medium (containing 1% penicillin-streptomycin) and 10 μL of MTT solution (5 mg/mL). Following a 4 h incubation, 100 μL of lysis buffer was added to solubilize the formazan crystals, and the plates were incubated for another 4 h. Absorbance readings were subsequently taken at 570 nm. The experiment was repeated three times (n = 3).

### 2.15. Live/Dead Staining

HUVECs were seeded in a 24-well plate and subjected to the respective treatments. After incubation for 24 or 48 h, the supernatant was removed, and 250 μL of Calcein AM/PI working solution was added to each well. The plate was then placed in a cell culture incubator for 30 min, followed by observation under a live-cell imaging system. A total of three replicates were performed for this experiment (n = 3).

### 2.16. Cell Scratch Assay

HUVECs were cultured in 12-well plates until they reached a confluent monolayer. Using a sterile 200 µL pipette tip, a scratch wound was made in each well. To remove any non-adherent cells and debris, the wells were washed gently with PBS (3–4 times). Subsequently, the cells were incubated with different media for the treatments. All experimental media were supplemented with 2% FBS and 1% penicillin-streptomycin, and consisted of: 1 mL of basal medium for the control group, 1 mL of blank hydrogel extract for the hydrogel group, and 1 mL of blank hydrogel extract containing 20 µM ADT-OH for the treatment group. Cell migration into the wound area was monitored and imaged at 0, 12, 24, and 48 h using an inverted fluorescence microscope. The experiment was repeated three times (n = 3). The wound area at each time point was quantified using ImageJ 1.53k software. The wound closure rate and/or cell migration rate were then calculated according to the specified Equation (4):

Cell migration (%) = (T_0_ − T_n_)/T_0_ × 100% (4)

where T_n_: scratch area at n hours, T_0_: scratch area at 0 h.

### 2.17. Transwell Migration Assay

HUVECs were seeded into the upper chamber of a Transwell insert. The lower chamber was filled with medium containing the respective test samples. After 24 h of incubation, cells in the upper chamber were gently rinsed with PBS and fixed with 4% paraformaldehyde for 10 min. Following washing, the cells were stained with 0.1% crystal violet for 10 min. Non-migrated cells on the upper surface of the membrane were carefully removed. Migrated cells on the lower surface were first observed under an upright microscope. For quantification, the stained cells were dissolved in acetic acid, and the absorbance of the solution was measured at 590 nm. The experiment was repeated three times (n = 3).

### 2.18. In Vitro ROS Scavenging Assay

HUVECs were seeded in a 24-well plate and co-incubated for 6 h with Rosup (50 μg mL^−1^, used as a ROS inducer) and the test compounds. Following incubation, the cells were washed twice with PBS. To detect intracellular reactive oxygen species, the cells were loaded with 10 μM DCFH-DA for 30 min. Cell nuclei were then counterstained with DAPI for visualization. Three independent biological replicates were performed (n = 3). Fluorescence images were acquired using a live-cell imaging system.

### 2.19. In Vitro Detection of Intracellular H_2_S

To detect intracellular hydrogen sulfide (H_2_S) levels, HUVECs seeded in confocal dishes were incubated for 6 h with hydrogel extract medium containing a 10 μM H_2_S fluorescent probe (HSP). After incubation, the cells were washed three times with PBS and stained with DAPI to label the nuclei. Three independent biological replicates were performed (n = 3). Images were acquired using a confocal laser scanning microscope (CLSM; Zeiss LSM 800, Carl Zeiss AG, Oberkochen, Germany).

### 2.20. Tube Formation Assay

To evaluate tube formation, 30 μL of chilled Matrigel was dispensed into each well of a cold 24-well plate. The plate was then placed at 37 °C for 30 min to allow the gel to solidify. After the respective treatments, HUVECs were plated onto the Matrigel layer and cultured for 6 h. Tubular structures were examined under an inverted microscope. Quantitative analysis of branching points and total tube length was performed using ImageJ software. A total of three replicates were performed for this experiment (n = 3).

### 2.21. In Vivo Study of ACMOD Hydrogel on Burn Wound Healing

Experimental animals were provided by Guangdong Medical Laboratory Animal Center (Guangzhou, China). Animal experiments were approved by the Experimental Animal Ethics Committee of Jinan University (20200314-07). To establish a composite burn wound model, 24 male Sprague-Dawley rats weighing 180~210 g were randomly divided into four groups, with 6 rats in each group. After anesthesia, the dorsal hair was shaved. Second-degree burns were created using an alkali scald method: a three-layer gauze pad (diameter 1.5 cm) saturated with 2.5 mol/L NaOH solution was applied with pressure to both sides of the rat’s back for 60 s [33]. Subsequently, a full-thickness skin defect with a diameter of 1.0 cm was excised using a biopsy punch. The rats were sacrificed on days 7 and 14 after treatment to evaluate both early and late stages of the wound healing process.

The animals were treated as follows: Control group (normal saline), CMOD group, ACMOD group, and 3M group. Wound areas were photographed on days 0, 3, 7, 11, and 14. The wound area at each time point was quantified using ImageJ software, and the wound contraction rate was calculated according to the following Formula (5):

Wound contraction rate (%) = (A_0_ − A_t_)∕A_0_ × 100% (5)

where A_0_: the wound area at day 0, A_t_: the wound area at day t.

### 2.22. Quantification of Inflammatory Cytokines

Fourteen days after treatment, the rats were euthanized, and the skin tissues from the wound periphery were harvested and homogenized. Tissue homogenates were then centrifuged at 4 °C, and the supernatants were collected. The levels of IL-10 (anti-inflammatory), TNF-α, and IL-6 (pro-inflammatory) were measured using corresponding commercial ELISA kits (Enzyme Linked Biotechnology Co., Ltd., Shanghai, China), strictly adhering to the manufacturer’s instructions. The study was performed with three biological replicates (n = 3).

### 2.23. Determination of Oxidative Stress Levels in Wound Tissue

On day 7 post-treatment, rats were euthanized, and skin tissues surrounding the wound area were collected. To assess the level of ROS, frozen tissue sections were prepared and stained with dihydroethidium (DHE) for 30 min, followed by nuclear counterstaining with DAPI. After washing, the sections were mounted and visualized under an inverted fluorescence microscope. The study was performed with three biological replicates (n = 3).

### 2.24. Immunofluorescence Staining of Macrophage Markers in Wound Tissue

Macrophage polarization in the wound region was evaluated via immunofluorescence staining for M1 (CD86) and M2 (CD206) markers, with three biological replicates (n = 3). Rats were sacrificed on post-treatment day 7, and the wound tissues with adjacent skin were harvested and fixed. Following paraffin embedding and sectioning, the tissue slides were deparaffinized, rehydrated, and subjected to heat-induced antigen retrieval. Subsequently, the sections were incubated with anti-CD86 and anti-CD206 primary antibodies, followed by appropriate fluorophore-labeled secondary antibodies. Nuclei were counterstained with DAPI. After washing and mounting, the slides were visualized using an inverted fluorescence microscope, and images were acquired to analyze the distribution patterns of CD86^+^ (M1) and CD206^+^ (M2) macrophages.

### 2.25. Immunofluorescence Staining of VEGF in Wound Tissue

To assess angiogenesis within the healed wound, VEGF expression was analyzed by immunofluorescence. The study was performed with three biological replicates (n = 3). On day 14 post-treatment, rats were euthanized, and the wound tissue along with adjacent skin was collected and fixed. Following paraffin embedding and sectioning, the tissue slides were deparaffinized, rehydrated, and subjected to antigen retrieval. The sections were then incubated with a primary antibody against VEGF, followed by a corresponding fluorescently labeled secondary antibody. Cell nuclei were counterstained with DAPI. After washing and mounting, the slides were examined under an inverted fluorescence microscope, and representative images were captured for qualitative and semi-quantitative analysis of VEGF expression.

### 2.26. Histological Analysis

At predetermined time points (days 7 and 14 after treatment), wound tissues including the central wound area and adjacent normal skin were harvested. The harvested specimens were fixed in 4% paraformaldehyde, dehydrated in a graded alcohol series, cleared in xylene, and infiltrated with paraffin before being embedded into blocks. Serial cross-sections, each 5 μm thick, were cut with a microtome. For morphological observation, sections were stained with hematoxylin and eosin (H&E); for collagen deposition assessment, adjacent sections were stained with Masson’s trichrome. All stained slides were visualized under a light microscope, and images were captured for subsequent quantitative or qualitative analysis.

### 2.27. In Vivo Safety Assessment

The potential systemic toxicity of the ACMOD hydrogel was investigated on day 14 after treatment. Rats were euthanized, and a complete necropsy was performed. Organs including the heart, liver, spleen, lungs, and kidneys were excised and fixed in 4% paraformaldehyde. These samples were subsequently processed, embedded in paraffin, sectioned, and stained with H&E for histopathological evaluation under a light microscope. For hematological analysis, blood specimens were collected into anticoagulant tubes containing EDTA-K2 and gently inverted to ensure adequate anticoagulation. Routine blood indices were then quantified using an automated hematology system.

### 2.28. Statistical Analysis

GraphPad Prism software (version 9.5) was employed to perform all statistical analyses. Quantitative data were expressed as the mean ± standard deviation (SD). For comparisons between two groups, Student’s *t*-test was applied, while one-way analysis of variance (ANOVA) followed by an appropriate post hoc test was used for multiple group comparisons. Statistical significance was defined as * *p* < 0.05, ** *p* < 0.01, *** *p* < 0.001, and **** *p* < 0.0001.

## 3. Results and Discussion

### 3.1. Synthesis and Characterization of OD and Hydrogels

**Figure 1:**
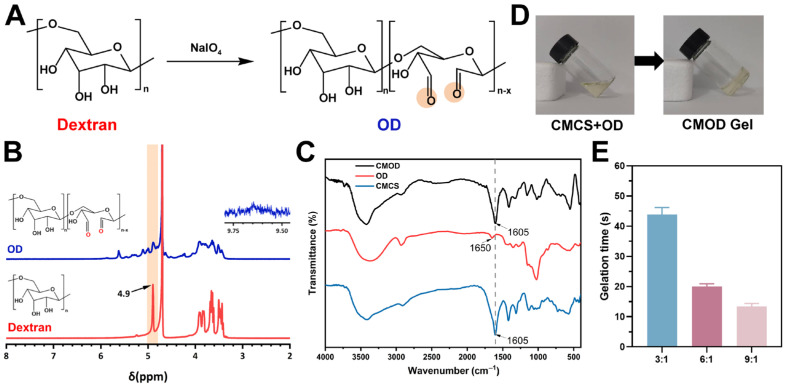
Preparation and characterization of the hydrogel. (**A**) Schematic illustration of the oxidation process of dextran by sodium periodate. (**B**) ^1^H NMR spectra of dextran and OD. (**C**) FTIR spectra of CMCS, OD, and the formed CMOD hydrogel. (**D**) Photograph showing the formation of the CMOD hydrogel. (**E**) Gelation time of hydrogels formed at different CMCS:OD ratios. Data are presented as mean ± SD (n = 3).

NaIO_4_ selectively cleaves the vicinal diols in dextran, generating dialdehyde groups to form OD, as illustrated in Figure 1A. It is worth noting that periodate oxidation may reduce the molecular weight of dextran due to potential main chain scission during the reaction [34]. The extent of chain degradation is influenced by factors such as periodate concentration, reaction time, and temperature [35,36]. Under the mild conditions employed in this study, any chain scission is expected to be minimal, allowing the polymer to retain sufficient chain length for effective network formation. Successful oxidation was confirmed by ^1^H-NMR spectroscopy. The disappearance of the peak at 4.9 ppm indicated the consumption of hydroxyl groups at the C2 and C3 positions, while the emergence of a characteristic aldehyde proton signal between 9.60 and 9.65 ppm confirmed the introduction of aldehyde functionalities (Figure 1B). Further evidence was obtained from FT-IR analysis (Supplementary Materials Figure S1). Although the spectra of dextran and OD were largely similar, a subtle but distinct peak appeared at approximately 1735 cm^−1^ in the OD spectrum, corresponding to the C=O stretching vibration of the aldehyde group. Concurrently, the broad O-H stretching band (3800–3000 cm^−1^) showed reduced intensity in OD, consistent with the conversion of hydroxyl to aldehyde groups. The oxidation degree of OD, quantified via the hydroxylamine hydrochloride titration method, was determined to be around 67%. The high oxidation degree (~67%) of OD is crucial as it provides a dense population of reactive aldehyde sites for crosslinking, enabling the formation of robust hydrogels with CMCS. These findings align with previous reports [37,38]. OD was synthesized by periodate oxidation of dextran, with sodium periodate added at an equimolar ratio relative to the anhydroglucoside units of dextran. This stoichiometric ratio was selected to achieve a moderate oxidation degree, ensuring sufficient aldehyde group generation for subsequent Schiff base crosslinking while minimizing excessive polymer chain degradation that can occur under harsher oxidation conditions.

The CMOD Hydrogel was subsequently formed by mixing OD solution with CMCS solutions at various ratios. Gelation time, defined as the point when the mixture no longer flowed upon vial inversion for 30 s, was measured. Successful hydrogel formation was visually confirmed (Figure 1D). All compositions gelled rapidly (Figure 1E). As expected from reaction kinetics, a higher CMCS concentration accelerated the Schiff base reaction between its amino groups and the aldehydes on OD, leading to shorter gelation times. The inverse relationship between CMCS concentration and gelation time is a classic manifestation of reaction kinetics [39], where increased reactant concentration drives faster network formation. This tunability allows for the optimization of injectability and in situ gelation speed—a critical feature for an adaptable wound dressing. The formulation with a CMCS:OD volume ratio of 6:1 exhibited a suitable gelation time for practical wound application and was therefore selected for all subsequent experiments.

FT-IR spectroscopy corroborated the crosslinking mechanism. The spectrum of the CMOD hydrogel (Figure 1C) showed a characteristic peak at around 1605 cm^−1^. This peak represents the overlapping absorption bands of the C=O from CMCS’s carboxyl groups and the newly formed C=N (imine bond) from the Schiff base reaction [40]. Notably, the distinct aldehyde C=O peak of OD near 1650 cm^−1^ was absent in the CMOD spectrum, confirming the consumption of aldehydes during hydrogel formation. The spectroscopic evidence therefore supports the successful synthesis of OD and its subsequent reaction with CMCS via dynamic imine bond formation, which is the foundation for the hydrogel’s subsequent self-healing and injectable properties.

### 3.2. Morphological and Rheological Properties of the Hydrogel

**Figure 2:**
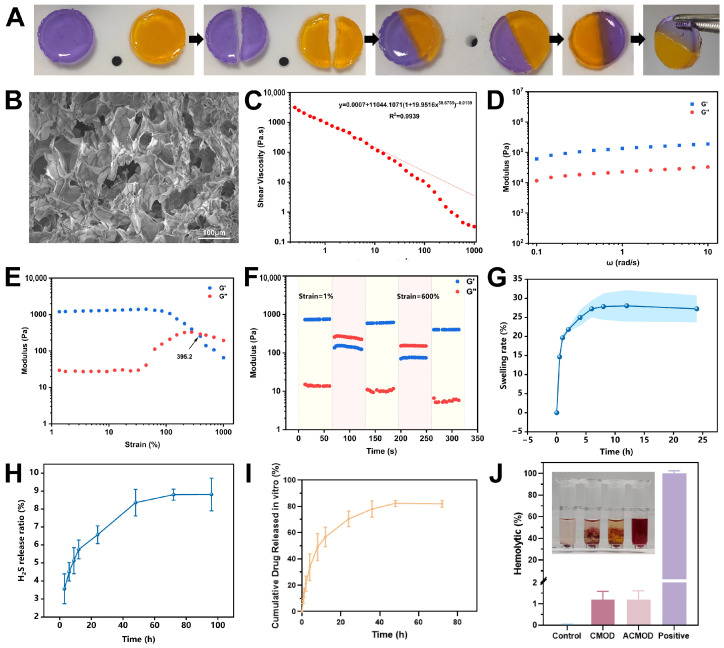
Characterization of hydrogel properties. (**A**) Macroscopic self-healing property of the CMOD hydrogel. (**B**) SEM image showing the internal microstructure of the CMOD hydrogel. Scale bars: 100 μm. (**C**) Steady shear viscosity measurement of the hydrogel. Red line shows the fitting curve. (**D**) Frequency sweep test of the hydrogel. (**E**) Amplitude sweep test of the hydrogel. (**F**) Step-strain test for evaluating the self-healing behavior. (**G**) Swelling kinetics of the hydrogel. (**H**) Cumulative H_2_S release profile from the ACMOD hydrogel. (**I**) In vitro cumulative release profile of ADT-OH from the ACMOD hydrogel. (**J**) Hemolysis assay of different hydrogels co-incubated with blood samples. The inset photograph illustrates the corresponding red blood cell suspensions after incubation. Data are presented as mean ± SD (n = 3).

A key requirement for wound dressings is the ability to withstand and recover from damage. Macroscopically, two differently dyed hydrogel halves, when placed in contact, seamlessly merged within two hours without external force and could be lifted without separation (Figure 2A), demonstrating excellent macroscopic self-healing. SEM revealed that the hydrogel possessed a uniform, porous three-dimensional network (Figure 2B). This microstructure is advantageous for absorbing wound exudate and facilitating potential drug delivery.

Rheological characterization provided deeper insights into the hydrogel’s mechanical behavior. It exhibited pronounced shear-thinning, with viscosity decreasing as shear rate increased from 0.1 to 1000 s^−1^ (Figure 2C). The fitting curve equation was y = 0.0007 + 11044.1071(1 + 19.9516x^58.6755^)^−0.0139^, R^2^ = 0.9939, confirming its non-Newtonian fluid nature and indicating injectability. Frequency sweep tests (0.1–10 rad/s) showed that the storage modulus (G′) consistently remained higher than the loss modulus (G″) with minimal variation across the tested frequencies (Figure 2D), indicating solid-like behavior and long-term stability. The LVR was identified via amplitude sweep. G′ dominated G″ up to a strain of approximately 395.2%, beyond which the structure yielded (Figure 2E). Most notably, alternating strain step tests between 1% (within LVR) and 600% (beyond LVR) demonstrated rapid and reversible recovery of G′ and G″ (Figure 2F). The network repeatedly broke down under high strain (G″ > G′) and fully recovered upon returning to low strain (G′ > G″), confirming excellent microscopic self-healing capability. This unique combination of properties is highly desirable for wound management: shear-thinning [41] enables painless, non-invasive injection to fill irregular wounds, while self-healing ensures the dressing maintains its protective barrier integrity against external friction or movement, prolonging its functional lifespan in vivo.

### 3.3. Swelling and Hydrogen Sulfide Generation Properties

The swelling ratio of the hydrogel reached equilibrium (~28%) within 8 h at 37 °C (Figure 2G). This moderate and rapid swelling capacity is critical for a wound dressing to effectively absorb excess exudate while maintaining a moist healing environment. Hydrogen sulfide (H_2_S) release from the ACMOD hydrogel was quantified using the methylene blue assay, calibrated with a sodium sulfide standard curve (Supplementary Materials Figure S2). The ACMOD hydrogel demonstrated sustained H_2_S release over 96 h, characterized by an initial rapid phase followed by a slower, sustained release that stabilized around 8% of the theoretical maximum (Figure 2H). This confirms the successful incorporation and activity of the H_2_S donor, ADT-OH, within the hydrogel matrix.

### 3.4. In Vitro Drug Release Profile

The release profile of ADT-OH from the ACMOD hydrogel was evaluated in PBS at 37 °C. As shown in Figure 2I, the release exhibited a biphasic pattern: an initial burst release of approximately 70% within the first 24 h, followed by a sustained, slower release over the next 48 h until reaching a plateau. These release kinetics correlate with the observed H_2_S generation profile. The interconnected porous structure observed via SEM facilitates fluid penetration and diffusion, explaining the initial rapid swelling and drug release. The subsequent sustained release phase is likely governed by a combination of Fickian diffusion [42] and gradual degradation/erosion of the hydrogel network. The biphasic release profile may be therapeutically advantageous: the initial burst could help establish a therapeutic concentration of H_2_S to rapidly modulate the early inflammatory phase of wound healing, while the sustained release ensures prolonged biological activity to support subsequent proliferation and remodeling phases.

### 3.5. Hemocompatibility Assessment

Hemolysis testing is a fundamental assay for evaluating the blood compatibility of biomaterials, serving as a critical indicator of both safety (cytotoxicity toward red blood cells) and hemocompatibility/stability of the formulation upon contact with blood. The hemolysis rates for both CMOD and ACMOD hydrogels were extremely low, at 1.19% and 1.21%, respectively (Figure 2J), well below the 5% safety threshold for biomedical materials. Visually, the supernatant from hydrogel-treated groups remained clear, in stark contrast to the deep red positive control (Triton X-100). The negligible hemolytic activity confirms the excellent blood compatibility of the hydrogel system. The low hemolysis rate indicates that the hydrogel and its degradation products do not cause significant damage to red blood cell membranes, significantly de-risking its potential for in vivo application and paving the way for subsequent biological evaluations. Furthermore, this result underscores the formulation’s stability, as the absence of hemolysis suggests that the hydrogel matrix remains intact and does not leach hemolytic components under physiological conditions.

### 3.6. In Vitro Biocompatibility and Cell Migration Assays

**Figure 3:**
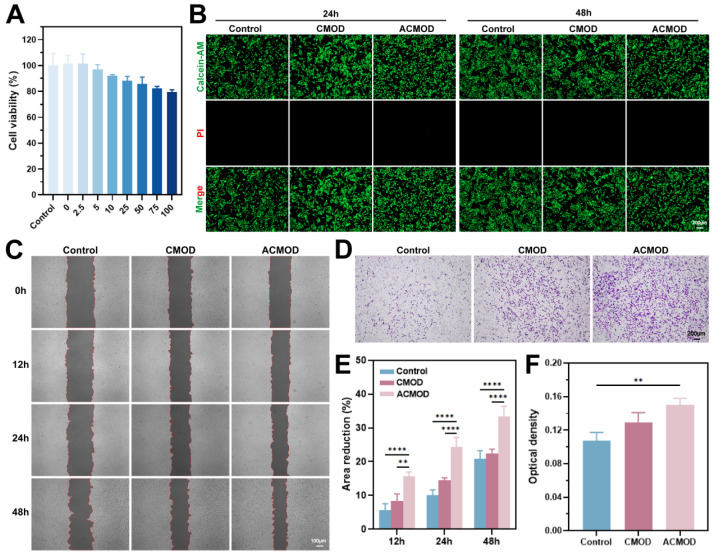
In vitro biocompatibility and promigratory effects of the hydrogels on HUVECs. (**A**) Viability of HUVECs after 24 h incubation with hydrogel extracts containing different concentrations of ADT-OH. (**B**) Live/dead staining of HUVECs after 24 h and 48 h incubation with hydrogel extracts (green: live cells; red: dead cells). Scale bars: 200 μm. (**C**) Representative images of the scratch wound healing assay at different time points after treatment with hydrogel extracts. Scale bars: 100 μm. (**D**) Transwell migration assay images of HUVECs after 24 h incubation with hydrogel extracts. Scale bars: 200 μm. (**E**) Quantitative analysis of cell migration rates from the scratch assay. (**F**) Quantitative analysis of migrated cells from the Transwell assay (crystal violet staining). Data are presented as mean ± SD (n = 3). ** *p* < 0.01, **** *p* < 0.0001.

The cytotoxicity of hydrogel extracts containing varying concentrations of ADT-OH was evaluated using the MTT assay. After 24 h of incubation, cell viability exhibited a concentration-dependent decrease with increasing ADT-OH levels, confirming that ADT-OH concentration influences cellular metabolic activity (Figure 3A). Notably, within the range of 0–100 μmol/L, cell viability remained above 80%, indicating no significant cytotoxicity and suggesting good biosafety for ADT-OH. In particular, cell viability remained above 85% at ADT-OH concentrations up to 50 μmol/L after 24 h of incubation with HUVECs. The excellent biocompatibility was further corroborated by live/dead staining (Figure 3B). After 24 h and 48 h of incubation, the majority of cells in both the CMOD and ACMOD groups were stained green (live), with only a minimal number appearing red (dead), similar to the control group. This indicates that the hydrogels themselves had no significant adverse effect on cell survival and exhibited excellent cytocompatibility in vitro.

A cell scratch test was employed to simulate cell migration during the early phase of wound repair. The ACMOD hydrogel significantly enhanced the migration of HUVECs compared to controls. After 24 h, the migration rate in the ACMOD group was 24.26%, substantially higher than that in the CMOD (14.41%) and control (10.06%) groups. This promotive effect was more pronounced after 48 h, with migration rates reaching 33.49% for ACMOD, compared to 22.47% for CMOD and 20.84% for the control (Figure 3C,E). The Transwell migration assay yielded consistent results. A marked increase in crystal violet-stained cells was observed in the ACMOD group (Figure 3D), which was confirmed quantitatively by absorbance measurement of the dissolved dye (Figure 3F). The significantly enhanced cell migration observed in both scratch and Transwell assays for the ACMOD group can be robustly attributed to the sustained release of H_2_S. H_2_S is a recognized gaseous signaling molecule known to promote cell migration and angiogenesis by modulating cytoskeletal dynamics, activating pro-migratory signaling pathways (e.g., PI3K/Akt), and enhancing cellular bioenergetics [43]. Enhanced cell migration is a pivotal step in wound healing, facilitating re-epithelialization and granulation tissue formation [44]. These compelling in vitro results strongly suggest that the ACMOD hydrogel, through its sustained H_2_S release, holds great potential to accelerate the cellular phase of burn wound healing in vivo.

### 3.7. In Vitro Antioxidant, H_2_S-Generating, Pro-Angiogenic and Anti-Inflammatory Properties

**Figure 4:**
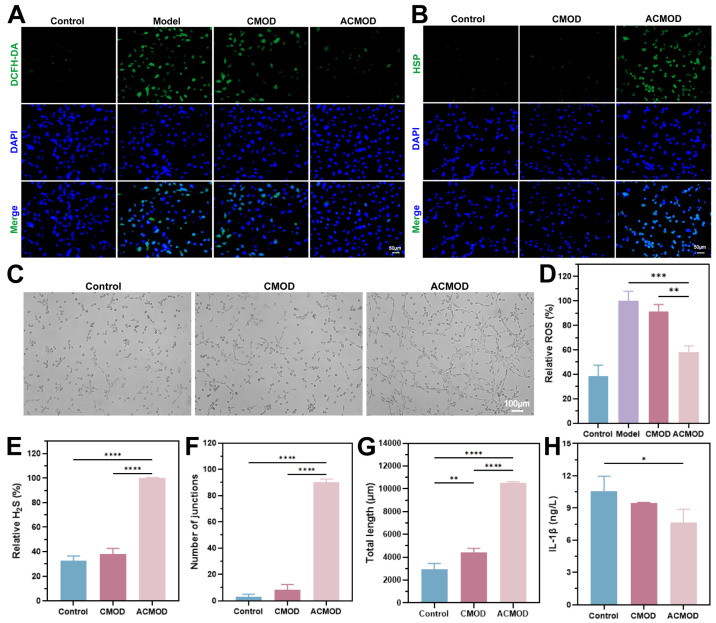
In vitro antioxidant, H_2_S-generating, pro-angiogenic, and anti-inflammatory properties of the hydrogels. (**A**) Representative fluorescence images showing intracellular ROS levels in HUVECs after different treatments. Scale bars: 50 μm. (**B**) Representative fluorescence images demonstrating intracellular H_2_S generation in HUVECs after different treatments. Scale bars: 50 μm. (**C**) Representative bright-field images of tube formation by HUVECs on Matrigel after 6 h of incubation with hydrogel extracts. Scale bars: 100 μm. (**D**) Semi-quantitative analysis of ROS fluorescence intensity. (**E**) Semi-quantitative analysis of H_2_S fluorescence intensity. (**F**,**G**) Quantitative analysis of the tube formation assay, showing the number of branching points and the total tube length. (**H**) Quantitative analysis of IL-1β secretion from HUVECs after different treatments, as measured by ELISA. Data are presented as mean ± SD (n = 3). * *p* < 0.05, ** *p* < 0.01, *** *p* < 0.001, **** *p* < 0.0001.

To evaluate the antioxidant capacity of the ACMOD hydrogel, intracellular reactive oxygen species (ROS) were induced in HUVECs using Rosup. Compared to the model (Rosup-only) group, the ACMOD treatment group showed a significant reduction in ROS-associated fluorescence intensity, demonstrating its potent ROS-scavenging ability (Figure 4A). Semi-quantitative analysis indicated that the ACMOD hydrogel reduced intracellular ROS levels by approximately 42% compared to the control (Figure 4D). Furthermore, the ability of the hydrogel to stimulate intracellular H_2_S production was assessed using the H_2_S-specific fluorescent probe (HSP). A marked increase in green fluorescence was observed in the ACMOD group (Figure 4B), and semi-quantification of the fluorescence intensity confirmed a statistically significant elevation compared to the control (Figure 4E), verifying the H_2_S-generating capability of ACMOD. The observed 42% reduction in ROS levels can be directly attributed to the well-documented antioxidant properties of H_2_S, which can act as a direct scavenger of reactive species and may upregulate endogenous antioxidant defense systems. This antioxidant effect is crucial for mitigating oxidative stress, a major impediment to normal wound healing that prolongs inflammation and damages cellular components [45].

The pro-angiogenic potential of the hydrogel was investigated via a tube formation assay. After 6 h of incubation, the ACMOD group exhibited a significantly more extensive and interconnected network of capillary-like structures compared to both the control and CMOD groups (Figure 4C). Quantitative analysis revealed that the ACMOD hydrogel led to a greater number of branching points (Figure 4F) and increased total tube length (Figure 4G).

Finally, the anti-inflammatory effect was evaluated by measuring the secretion of the key pro-inflammatory cytokine IL-1β. ELISA results showed that the IL-1β level in the ACMOD group was significantly lower than that in the control group (Figure 4H), indicating the hydrogel’s ability to suppress pro-inflammatory cytokine production. Chronic inflammation is a hallmark of non-healing wounds. H_2_S has been shown to modulate immune responses, often by inhibiting the activation of the NLRP3 inflammasome, a key complex responsible for IL-1β maturation [46]. By reducing this pivotal pro-inflammatory cytokine, the ACMOD hydrogel helps shift the wound microenvironment from a pro-inflammatory state to a pro-regenerative one.

In summary, these in vitro results collectively demonstrate that the ACMOD hydrogel, through the controlled release of H_2_S, concurrently executes three complementary wound-healing-promoting actions: neutralizing harmful ROS, stimulating the growth of new blood vessels, and dampening excessive inflammation. This tripartite mechanism strongly supports its therapeutic potential for accelerating the repair of complex burn wounds.

### 3.8. In Vivo Evaluation of Burn Wound Healing

**Figure 5:**
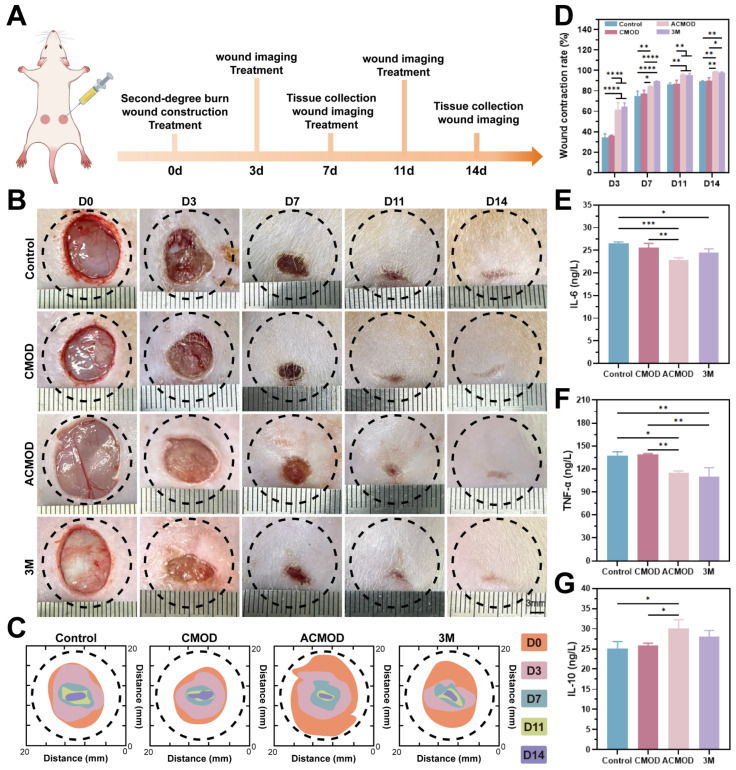
In vivo therapeutic efficacy of the ACMOD hydrogel on burn wound healing. (**A**) Schematic diagram of the burn wound treatment protocol. (**B**) Representative photographic images of wounds at different time points after various treatments. Scale bars: 3 mm. (**C**) Schematic representation of the wound healing progression across different treatment groups. (**D**) Quantitative analysis of the wound closure rate at designated time points. (**E**–**G**) Expression levels of inflammatory cytokines (IL-6, TNF-α, and IL-10) in wound tissues collected on day 14 post-treatment, as measured by ELISA. Data are presented as mean ± SD (n = 3). * *p* < 0.05, ** *p* < 0.01, *** *p* < 0.001, **** *p* < 0.0001.

To evaluate the therapeutic effect of the ACMOD hydrogel on burn wounds, a composite burn wound model was established in rats, as outlined in the experimental scheme (Figure 5A). The wounds were treated with PBS (control), CMOD hydrogel, ACMOD hydrogel, or a commercial 3M film. Wound healing was photographed and monitored on days 0, 3, 7, 11, and 14 post-treatment (Figure 5B). As early as day 3, the ACMOD-treated group exhibited markedly enhanced healing, with a wound area reduction of approximately 61.79%, significantly greater than the 34.32% reduction observed in the control group. This early advantage can be attributed to the timely pharmacological actions of the sustained H_2_S release. During the critical inflammatory phase, H_2_S likely mitigated initial oxidative stress and excessive inflammation—key barriers to healing—thereby facilitating a quicker transition to the proliferative phase. Although wound area gradually decreased in all groups over time, the ACMOD group consistently maintained a significantly higher healing rate than both the control and CMOD groups on days 7 and 11. By day 14, the wound contraction rates reached 89.12% (control), 89.98% (CMOD), 98.77% (ACMOD), and 97.93% (3M) (Figure 5C,D). The ACMOD group achieved near-complete wound closure, demonstrating the most effective healing outcome. These results indicate that ACMOD is not merely a protective cover but an active therapeutic agent. Its mechanism extends beyond physical protection to include biochemical modulation of the wound bed.

Furthermore, to assess the inflammatory response within the healed wound, tissue samples were collected on day 14 for cytokine analysis. ELISA results showed that the ACMOD treatment significantly downregulated the expression of the pro-inflammatory cytokines IL-6 (Figure 5E) and TNF-α (Figure 5F), while concurrently upregulating the anti-inflammatory cytokine IL-10 (Figure 5G). This indicates that the ACMOD hydrogel effectively modulated the wound microenvironment towards an anti-inflammatory and pro-regenerative state.

### 3.9. Immunofluorescence Analysis of Wound Tissues

**Figure 6:**
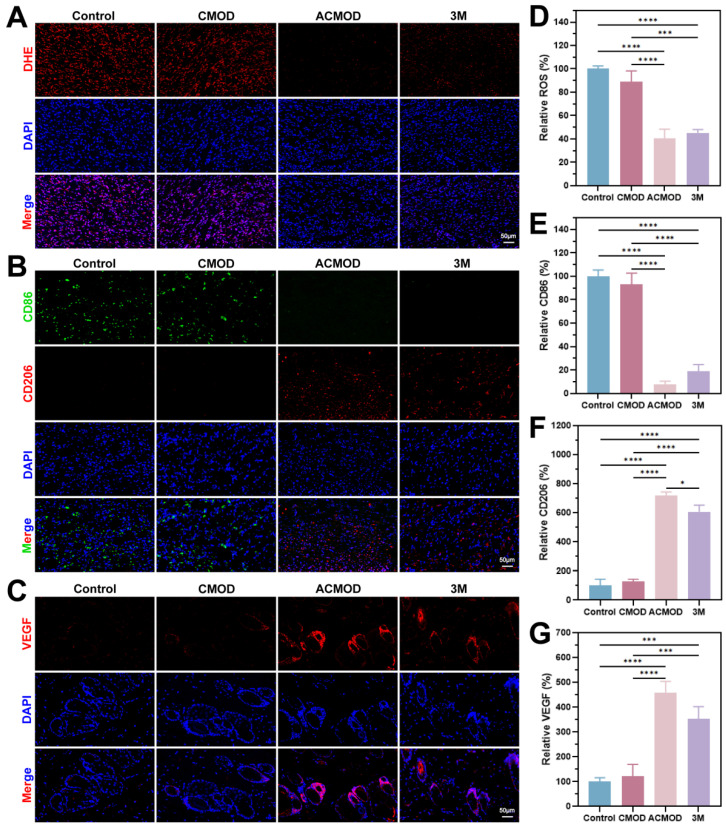
Mechanisms of ACMOD hydrogel action in promoting burn wound healing in vivo. (**A**) Representative images of DHE staining showing ROS levels in wound tissues on day 7 post-treatment. Scale bars: 50 μm. (**B**) Representative immunofluorescence images of wound tissues stained for the macrophage markers CD86 and CD206 on day 7. Scale bars: 50 μm. (**C**) Representative immunofluorescence images showing VEGF expression in wound tissues on day 14. Scale bars: 50 μm. (**D**) Semi-quantitative analysis of the DHE fluorescence intensity. (**E**,**F**) Semi-quantitative analysis of the fluorescence intensity for CD86 and CD206. (**G**) Semi-quantitative analysis of the VEGF fluorescence intensity. Data are presented as mean ± SD (n = 3). * *p* < 0.05, *** *p* < 0.001, **** *p* < 0.0001.

To elucidate the mechanisms underlying the accelerated healing promoted by the ACMOD hydrogel, a series of immunofluorescence and histological analyses were performed on wound tissues at strategically selected time points corresponding to key phases of the healing cascade. Day 7 post-wounding represents the transition from the inflammatory to the proliferative phase, providing an optimal window to assess early healing events. At this time point, in vivo ROS levels at the wound site were assessed using DHE staining. Consistent with the in vitro findings, the ACMOD-treated group exhibited a significant reduction in red fluorescence intensity compared to the control group (Figure 6A,D). This confirms that the sustained release of H_2_S from ADT-OH effectively scavenges excessive ROS in the wound microenvironment, thereby mitigating oxidative stress—a key contributor to tissue damage and delayed healing. Macrophages play a pivotal role in coordinating the inflammatory and proliferative phases of healing [47]. Immunofluorescence staining for the classical M1 marker CD86 (pro-inflammatory) and the M2 marker CD206 (anti-inflammatory) was also conducted on day-7 tissues. The ACMOD treatment significantly downregulated CD86 expression while upregulating CD206 expression compared to the control (Figure 6B,E,F). This shift in macrophage phenotype from M1 to M2 indicates that H_2_S actively reprograms the local immune response, fostering an anti-inflammatory and pro-regenerative microenvironment conducive to tissue repair. Overall, the rapid mitigation of oxidative stress and the strategic modulation of inflammation work synergistically to resolve the hostile early wound environment. By quenching free radicals and suppressing prolonged pro-inflammatory signaling, ACMOD creates a permissive landscape for regeneration, setting the stage for subsequent proliferative events.

Day 14 post-wounding corresponds to the peak of the proliferative phase, characterized by active granulation tissue formation, angiogenesis, and tissue remodeling. To evaluate whether the early immunomodulatory effects translated into enhanced tissue regeneration, we assessed angiogenic activity at this time point. The expression of vascular endothelial growth factor (VEGF), a master regulator of angiogenesis, was evaluated by immunofluorescence on day 14. A pronounced increase in VEGF signal was observed in the ACMOD group compared to control (Figure 6C,G). This upregulation is likely a direct consequence of H_2_S activity, which is known to activate pro-angiogenic signaling pathways, thereby stimulating endothelial cell function and new blood vessel formation [48]. Collectively, these findings demonstrate that ACMOD promotes burn wound healing through a dual mechanism: first, by resolving oxidative stress and inflammation during the early phase (day 7) to establish a regenerative microenvironment, and second, by enhancing angiogenesis during the proliferative phase (day 14) to support robust tissue regeneration.

### 3.10. Histological Analysis of Wound Tissues

**Figure 7:**
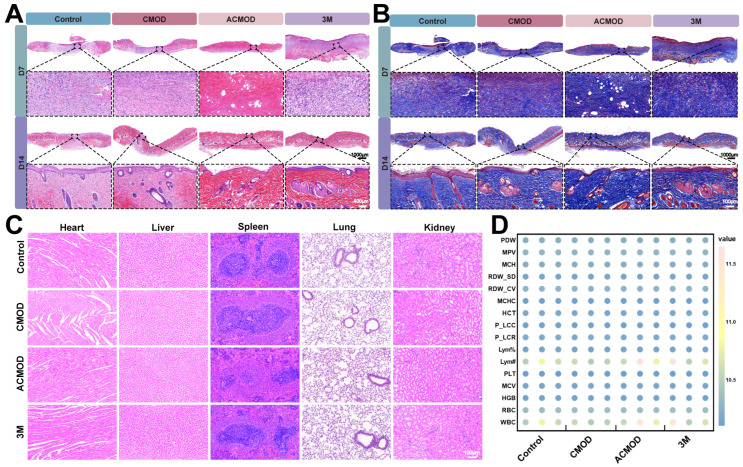
Histological evaluation of wound tissue regeneration. (**A**) Representative H&E-stained sections of wound tissues harvested on days 7 and 14 post-treatment. (**B**) Representative Masson’s trichrome-stained sections of wound tissues from the same time points. Collagen fibers are stained blue, demonstrating the extent and organization of collagen deposition during the healing process. The upper panels show overviews of the wound area (scale bars: 1000 μm). The lower panels display magnified views of the boxed regions (scale bars: 100 μm). (**C**) Representative H&E-stained images of major organs from SD rats on day 14 post-treatment. Scale bars: 100 μm. (**D**) Hematological analysis of SD rats on day 14 post-treatment.

H&E and Masson’s trichrome staining were performed on days 7 and 14 to assess tissue regeneration and collagen deposition. On day 7, the ACMOD group exhibited the shortest wound gap, the thickest nascent epithelium, and the most robust formation of granulation tissue (Figure 7A). By day 14, wounds in the ACMOD group were nearly fully re-epithelialized with restored skin appendage structures. Masson’s staining revealed dense, well-organized, and abundant collagen fibers (stained blue) in the ACMOD group, significantly exceeding that in other groups (Figure 7B). Collagen, the primary structural protein of the extracellular matrix, provides tensile strength and a scaffold for cell migration [49]. Its accelerated and orderly deposition under ACMOD treatment underscores the quality and maturity of the healed tissue. Therefore, the dual action of promoting VEGF expression and enhancing collagen synthesis addresses the core requirements of the proliferative and remodeling phases. VEGF-driven angiogenesis ensures a sustained supply of oxygen and nutrients, which fuels the metabolic demands of proliferating fibroblasts and keratinocytes [50]. The consequent high-quality, well-organized collagen deposition directly translates into superior tensile strength and reduced risk of scar formation. The blank CMOD hydrogel, lacking H_2_S, failed to elicit these profound cellular and molecular responses, highlighting that the physical matrix primarily provides a passive scaffold, while the bioactive H_2_S component is the key driver of healing dynamics.

Systemic safety is paramount for any therapeutic material. Histopathological examination of major organs (heart, liver, spleen, lungs, kidneys) showed normal architecture with no signs of lesions, degeneration, or inflammatory infiltration (Figure 7C). Furthermore, hematological analysis revealed no significant differences in key parameters (e.g., white/red blood cell counts, hemoglobin, platelets) between ACMOD-treated rats and healthy controls (Figure 7D). These results collectively confirm the excellent systemic biocompatibility and lack of overt toxicity of the ACMOD hydrogel.

## 4. Conclusions

This study successfully developed a self-healing hydrogel system (ACMOD) for advanced full-thickness burn wound management. The hydrogel was formed via a dynamic Schiff base reaction between OD and CMCS. By incorporating the H_2_S donor ADT-OH, ACMOD hydrogel was endowed with sustained hydrogen sulfide release. This design integrates the favorable physical properties of a dynamic hydrogel—including shear-thinning injectability, rapid self-healing, exudate absorption, and maintenance of a moist wound environment—with the multifaceted therapeutic functions of H_2_S gas therapy. Comprehensive in vitro and in vivo evaluations demonstrated the pronounced efficacy of ACMOD. In a rat model, ACMOD significantly accelerated wound closure and achieved near-complete healing by day 14. Mechanistically, the enhanced repair was orchestrated through multiple H_2_S-mediated processes: (1) scavenging excessive reactive oxygen species (ROS) to mitigate oxidative stress; (2) reprogramming macrophage polarization from a pro-inflammatory M1 to an anti-inflammatory M2 phenotype to resolve persistent inflammation; (3) upregulating VEGF expression to stimulate angiogenesis; and (4) promoting dense and well-organized collagen deposition to support high-quality tissue regeneration.

In summary, this work presents a promising strategy that combines a dynamic, user-friendly wound dressing with the pleiotropic biological activities of a gaseous mediator. The multifunctionality enabled by controlled H_2_S release represents a meaningful advance beyond conventional passive dressings for the treatment of challenging full-thickness burn wounds.

## Data Availability

The original contributions presented in this study are included in the article/Supplementary Materials. Further inquiries can be directed to the corresponding author.

## Supplementary Materials

The following supporting information can be downloaded at: https://www.mdpi.com/article/10.3390/pharmaceutics18030370/s1, Figure S1: FTIR spectra of dextran and OD. Figure S2: Standard curve of sodium sulfide for H_2_S quantification.
