# Supplementary material for: Oxidized Dextran/Carboxymethyl Chitosan Dynamic Schiff-Base Hydrogel for Sustained Hydrogen Sulfide Delivery and Burn Wound Microenvironment Remodeling
Source: Pharmaceutics. 2026 Mar 17;18(3):370. doi: 10.3390/pharmaceutics18030370 (PMC13030501; doi:10.3390/pharmaceutics18030370)
Supplement: Supplementary file 1 [file pharmaceutics-18-00370-s001.zip › pharmaceutics-4165833-supplementary.pdf]

Oxidized Dextran/Carboxymethyl Chitosan Dynamic Schiff-Base Hydrogel for Sustained Hydrogen Sulfide Delivery and Burn Wound Microenvironment Remodeling

Zhishan Liu [1,2,3,†], Ying Zhu [2,3,†], Zhuoya Ma [1,2,3], Xuyang Ning [1,2,3], Ziqiang Zhou [1,2,3], Jinchang Liu [1], Youfu Xie [1], Gang Li [1], Ping Hu [1,2,3,*]

[1] *Department of Burns & Plastic Surgery, Guangzhou Red Cross Hospital, Faculty of Medical Science, Jinan University, Guangzhou 510006, China*
[2] *State Key Laboratory of Bioactive Molecules and Druggability Assessment, Jinan University, Guangzhou 510006, China.*
[3] *College of Pharmacy, Jinan University, Guangzhou 510006, China.*

[†]: These authors contributed equally.
Corresponding author: Ping Hu*
Mailing address: Jinan University, No.855, East Xingye Avenue, Panyu Districst, Guangzhou 510632, CHINA
Tel.: +86-18581483142
E-mail: inzahu@hotmail.com

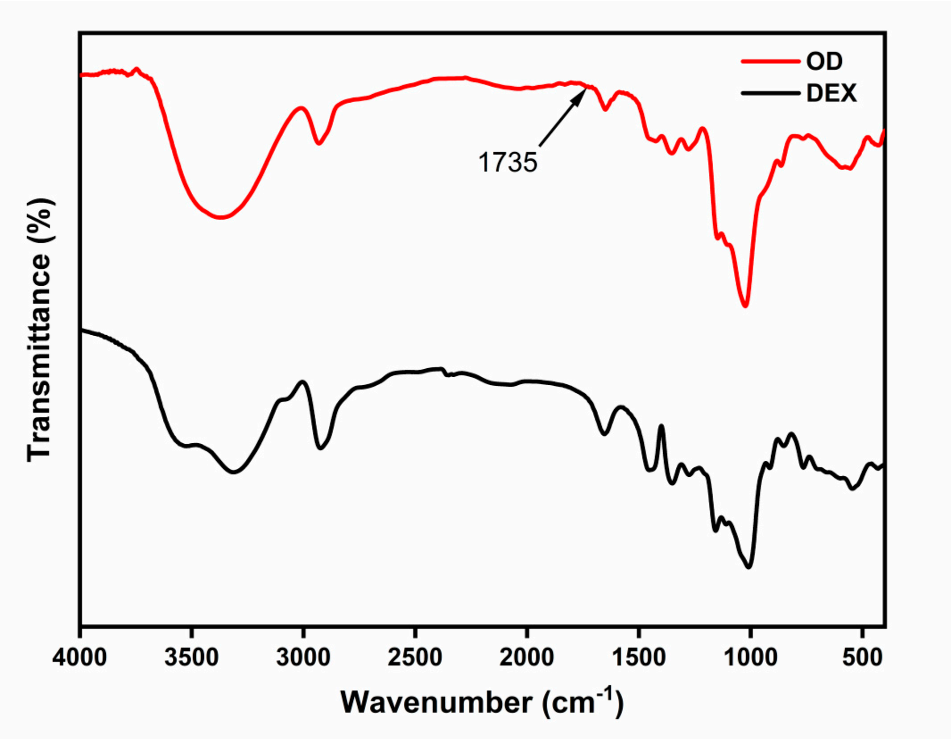

Figure S1. FTIR spectra of dextran and OD.

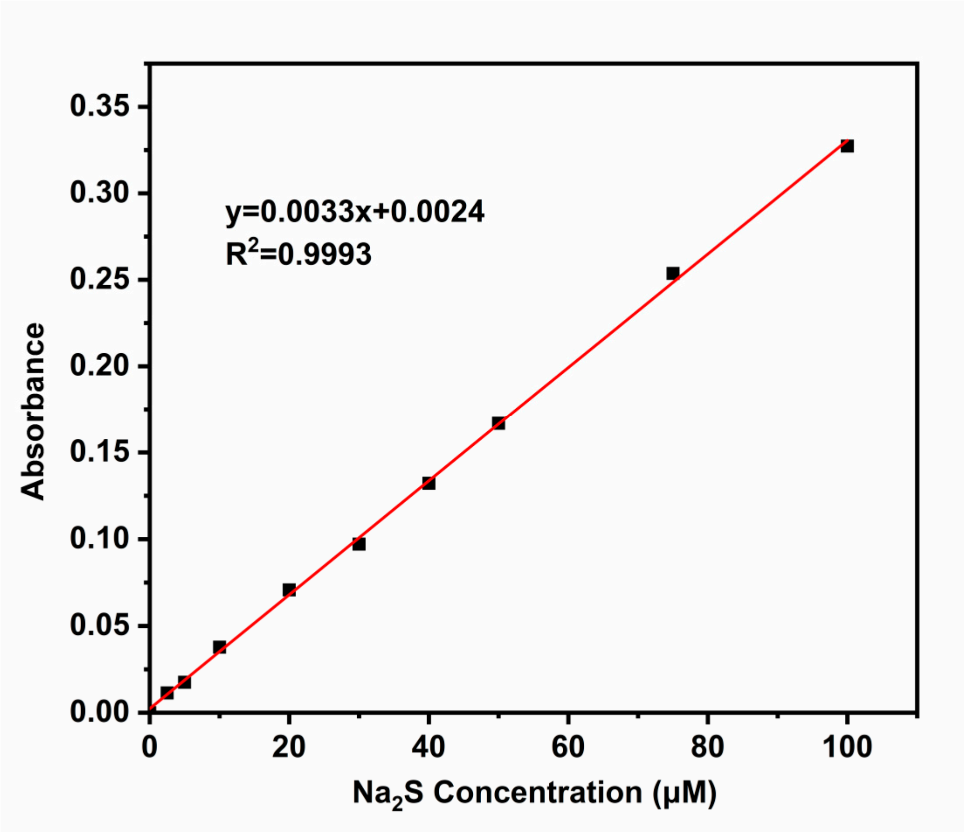

Figure S2. Standard curve of sodium sulfide for H_2S quantification.
